# Involvement of Inheritance in Determining Telomere Length beyond Environmental and Lifestyle Factors

**DOI:** 10.14336/AD.2023.1023

**Published:** 2023-11-05

**Authors:** Naheemat Modupeola Gold, Michael Ngozi Okeke, Yonghan He

**Affiliations:** ^1^Key Laboratory of Healthy Aging Research of Yunnan Province, Kunming Institute of Zoology, Chinese Academy of Sciences, Kunming 650201, Yunnan, China.; ^2^State Key Laboratory of Genetic, Evolution and Animal Models, Kunming Institute of Zoology, Chinese Academy of Sciences, Kunming 650201, Yunnan, China.; ^3^University of Chinese Academy of Sciences, Beijing 100049, China.; ^4^Center for Nanomedical Technology Research, Shenzhen Institute of Advanced Technology, Chinese Academy of Sciences, Shenzhen 518055, China.

**Keywords:** aging, age-related disease, determinant, inheritance, telomerase, telomere length

## Abstract

All linear chromosomal ends have specific DNA-protein complexes called telomeres. Telomeres serve as a "molecular clock" to estimate the potential length of cell replication. Shortening of telomere length (TL) is associated with cellular senescence, aging, and various age-related diseases in humans. Here we reviewed the structure, function, and regulation of telomeres and the age-related diseases associated with telomere attrition. Among the various determinants of TL, we highlight the connection between TL and heredity to provide a new overview of genetic determinants for TL. Studies across multiple species have shown that maternal and paternal TL influence the TL of their offspring, and this may affect life span and their susceptibility to age-related diseases. Hence, we reviewed the linkage between TL and parental influences and the proposed mechanisms involved. More in-depth studies on the genetic mechanism for TL attrition are needed due to the potential application of this knowledge in human medicine to prevent premature frailty at its earliest stage, as well as promote health and longevity.

## 1. Introduction

Telomeres are the structural DNA of a repetitive nucleotide (TTAGGG) and several associated protective proteins at the end of a linear chromosome [1]. Their primary function is to prevent the chromosomal end from being detected as a DNA double-strand break, which would cause the improper activation of the DNA damage repair machinery, leading to chromosome fusion and genomic instability during cell mitosis [2]. Hence, the loss of the protective telomeric capping function at the DNA end of the chromosome makes it susceptible to degradation and END-END fusion [3-5]. DNA polymerase, the enzyme responsible for DNA replication, cannot copy the nucleotides on each end of linear chromosome [6, 7]. When the RNA primer in the lagging strand of DNA is degraded and destroyed, a gap will remain in the DNA even after the RNA primer hybridizes to the exact chromosome, leading to the 3' end being longer than the 5' end [8, 9]. Thus, other mechanisms are needed to complete the replications at the linear DNA chromosome ends [10].

Cells may enter replicative arrest or die when telomeres reach critical lengths [11]. Telomere attrition can cause genomic instability, an important hallmark of aging [12]. Telomere length (TL) has been extensively studied as a biomarker of human aging and some studies reveal that its shortening is associated with lifespan [13]. TL is affected by several factors, which include the environment, nutrition, lifestyle, and genetics. For example, green spaces and neighborhood disorder risks have been reported as environmental factors that correlate with TL [14, 15]. Some studies suggest genetics as a contributing factor of the TL; individuals may inherit short or long telomeres from their parents, which could affect their life span and susceptibility to age-related disease [16-18]. In addition to human studies, evidence from several other organisms demonstrates a relationship between fitness and TL in early life, although no conclusive evidence has been found about the genetic basis of TL variation in wild animal populations due to high environmental variation compared to the laboratory [19].

In this review, we looked for original studies that provided pertinent data on factors that influence TL, with a focus on genetic factors, as to whether or not the length is paternally or maternally inherited in humans and other animal models. Understanding the genetic factors that influence TL can provide useful information on the initial setting of TL and programming of longevity in early life. This knowledge can help identify modifiable factors that can be targeted to prevent *in utero* telomere attrition and to improve health and longevity in offspring. Considering the scarcity of study protocols on this topic and the urgent necessity to clarify the mechanisms underlying senescence and telomere attrition, we herein present a review of the most recent inheritance studies on TL.

**Figure 1.**
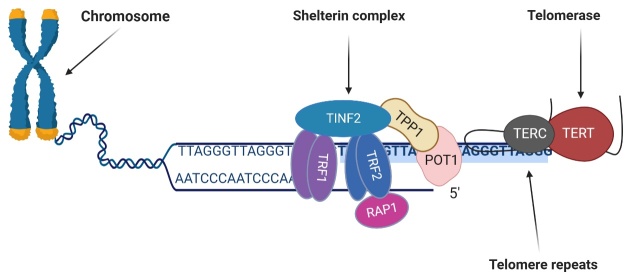
**Structure of telomere and telomerase**. Telomere is composed of the shelterin complex and the repetitive DNA sequence TTAGGG. Telomerase extends the length of shortened telomeres after DNA replication using TERC. TRF1, telomeric repeat binding factor 1; TRF2, telomeric repeat binding factor 2; POT1, protection of telomeres 1; TPP1, tripeptidyl peptidase 1; TINF2, TERF1 interacting factor 2; RAP1, repressor activator protein 1; TERC, telomerase RNA component; TERT, telomerase reverse transcriptase.

## 2. Telomere structure and function

The word 'telomere' comes from the Greek words Telos, meaning 'end', and Meros, meaning 'part'. Thus, telomeres refer to the nucleoprotein structures found at each chromosome end in eukaryotes [20]. Barbara McClintock first hypothesized, based on her studies on maize, that the telomeres at the ends of chromosomes protect them from degradation and fusion [21].

### 2.1 Telomere structure

Telomeres are composed of the shelterin complex, a collection of linked proteins, and a non-protein-coding, repetitive DNA sequence (TTAGGG) [22, 23]. An important function of the nucleoprotein complex is to protect the genetic material at the ends of the chromosome from shortening caused by DNA replication and stressful events [2, 24]. Six nucleoprotein-subunits, including telomeric repeat binding factor (TRF1), telomeric repeat binding factor 2 (TRF2), protection of telomeres 1 (POT1), tripeptidyl peptidase 1 (TPP1), TERF1 interacting factor 2 (TINF2), and repressor activator protein 1 (RAP1), make up the shelterin-nucleoprotein complex [25]. TRF1 and TRF2 are the molecular platforms that attract other shelterin factors and DNA repair proteins to telomeres [25, 26]. Ataxia telangiectasia mutated (ATM) and Ataxia telangiectasia and Rad3-related (ATR) kinases, the MRE11/RAD50/NBS1 complex, and the nuclease Apollo interact with TRF2, while the bloom syndrome helicase (BLM), the ATM, and DNA-dependent protein kinase catalytic subunit (DNA-PKcs) kinases interact with TRF1 [25]. These are specifically linked to mammalian telomeres and are crucial in protecting telomeres. The shelterin complex enables cells to discriminate between DNA damage and chromosomal ends. Deficiency in the nucleoprotein complex may result in telomere dysfunction, DNA damage, and cellular senescence [27]. Shelterin is also essential for the formation of the T-loop, a structure that blocks DNA damage response (DDR) caused by double-stranded breaks at chromosome ends, thereby inhibiting cellular aging, genomic instability and cancer [28, 29]. The structures of telomere, shelterin complex and telomerase are shown in Figure 1.

Telomere has a high G content (the single-stranded telomere G overhang) and can form a G quadruplex. This complex can contribute to and receive hydrogen bonds that keep the telomere stable [30]. The extended 3' G-rich end overhang folds back into the DNA duplex to form the T-loop. These factors make telomeres crucial for the integrity of eukaryotic genomes and the endurance of cellular information.

### 2.2 Function of telomere

One of the main functions of the telomere is to prevent chromosomal degradation and maintain stability. The telomere plays a crucial role in protecting the ends of chromosomes from DNA damage and unintentional recombination that occur when DNA end-joining takes place, which can lead to unintended consequences such as deletions, insertions, and substitution of nucleotides [31, 32]. Studies have shown that the inability of DNA polymerase in human cells to fully replicate the 3′ ends of the DNA strand during cell division, results in the shortening of the telomere by 30 to 200 base pairs over time due to a loss of the bases at the newly synthesized 5′ end [33]. This phenomenon, known as the end replication problem, leads to a gradual decrease in TL as people age. Human blood leukocyte TL (LTL) is known to shorten by 20 - 40 base pairs per year [34]. It is worth noting that before the telomere gets to a critical shorter length, a normal human cell can divide 50 - 70 times, which is known as the Hayflick limit [35].

Telomeres have another crucial function. For each cell cycle, a loss of nucleotide base pairs occurs during DNA replication, and when telomeres reach a certain length, the cell escapes the cell cycle, triggering senescence or apoptosis [36]. Yet, a small fraction of cell types (germ cells, cancer cells, and stem cells) can reverse increasing telomere shortening by activating the so-called "Telomere Maintenance Mechanisms (TMMs)" that can preserve TL [37]. Telomerase involvement in telomere maintenance is often mediated by telomerase activity or more rarely through combinatorial events, as in alternative lengthening of telomere (ALT) [38]. Telomeres can affect the expression of sub-telomeric genes by altering the architecture of adjacent chromatin through the telomere position effect (TPE). Although the mechanism of TPE is not fully understood, the effect of the TPE mechanism appears to be influenced by TL, local chromatin structure, and distance from the telomere [39].

### 2.3 Telomere regulation

Telomere is regulated by four factors: telomerase, the shelterin protein complex, telomeric repeat-containing RNA (TERRA) and CST (CTC1, STN1 and TEN1) complex. Telomerase is a ribonucleoprotein enzyme that synthesizes and maintains telomeres by using its own RNA component as a template [40]. However, the activity of telomerase can be modulated by other binding partners, such as transcription factors and signaling molecules, e.g, UV radiation, calcium, zinc, interferon α, estrogen, and transforming growth factor beta (TGF-beta) [41-43]. Additionally, telomerase directly contributes to cellular metabolism, signaling, and the control of gene expression in genes such as epidermal growth factor receptor (EGFR), vascular endothelia growth factor (VEGF) and nuclear factor-kappa B (NF-κB) dependent target genes in ways that are crucial for carcinogenesis, revealing telomere-independent regulation of cancer cell proliferation [44]. The two main components of human telomerase are telomerase reverse transcriptase (TERT) and telomerase RNA component (TERC), which serve as templates for telomere lengthening [45]. Using its own RNA template, telomerase replaces the missing base pairs that DNA polymerase does not replicate during transcription [46]. Most somatic cells exhibit little to no telomerase activity except for a small subset of human somatic cells (for example white blood cells), germ cells, and stem cells (for example, active fibroblasts) [43, 46]. In these cells, shortening of the telomere results in higher telomerase activity through telomere homeostasis [13]. Peripheral blood mononuclear cells (PBMCs) are a type of white blood cell that can be used to evaluate telomerase activity over a short time period, typically hours unlike T-lymphocytes, which require months to observe changes in telomerase activity. PBMCs show little telomerase expression, making it possible to evaluate it over a short time and show immediate, short-term alterations [47]. The role of shelterin and its structural components in the regulation of telomeres as discussed above, is mainly to protect telomeres. TERRA is a long non-coding RNA that is transcribed from the sub-telomeric regions of chromosomes [48]. It interacts with both telomeric DNA and proteins, and plays a crucial role in regulating TL, structure, and function [49]. TERRA can inhibit telomerase activity through base-pairing of the tandem repeats found throughout TERRA's 3'-end to the complementary RNA template region of telomerase [50], promote heterochromatin formation at chromosome ends by recruiting heterochromatin protein 1 (HP1) to telomeres [50], recruit DNA repair factors such as RAD51 and BRCA1 to telomeres, and modulate telomere recombination [48]. CST is a multiprotein complex that helps maintain the length and stability of telomeres [51]. This protein is composed of three subunits: CTC1, STN1, and TEN1 in higher organisms such as mammals and CDC13, STN1, and TEN1 in yeast [52]. CST is primarily located in single-stranded telomere DNA and plays an important role in preventing telomere overstretching [37]. Telomere overstretching occurs when telomeres become too long as a result of the overactivity of telomerase. Overstretched telomeres can inhibit telomerase activity and lead to cell senescence [51].

### 2.4 The biochemical pathway of telomerase lengthening of telomere

**Figure 2.**
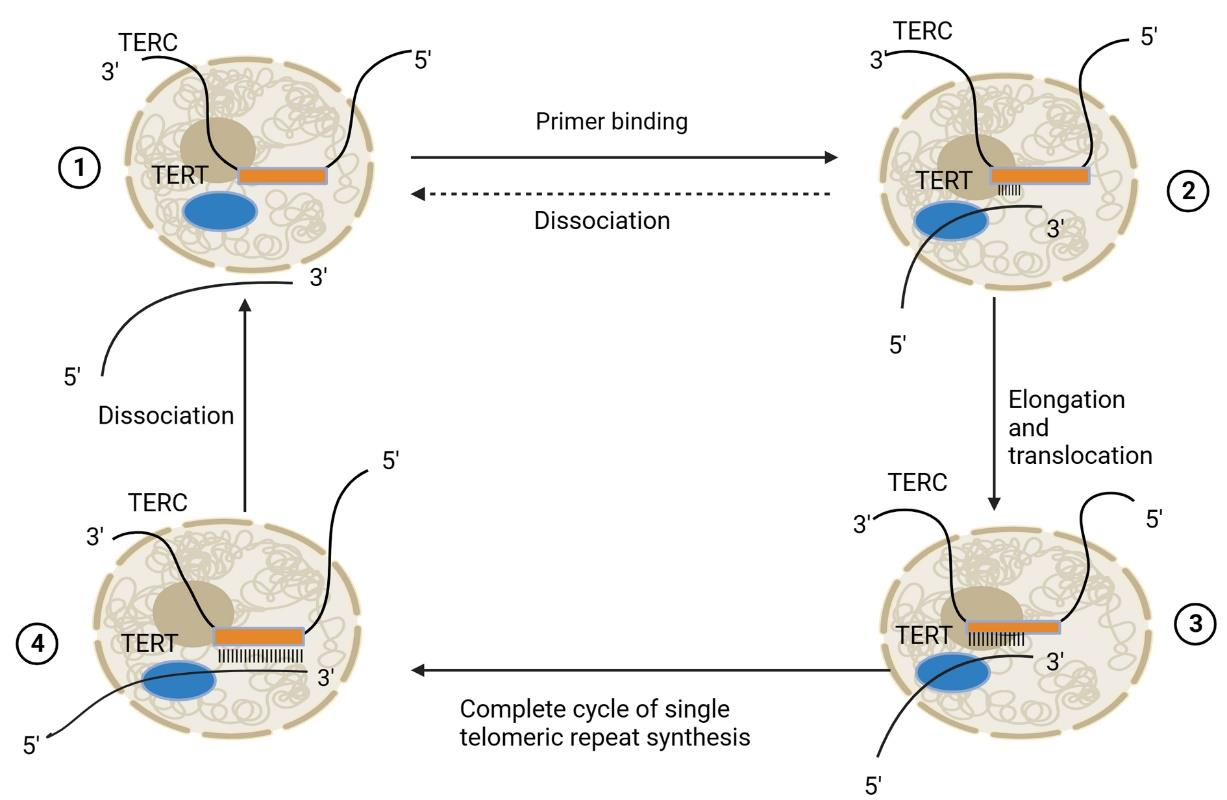
**Mechanism of telomerase pathway**. The mechanism of telomerase activity is illustrated in four stages: 1. primer not bound to TERT; 2. primer binding to TERT and annealing to TERC; 3. elongation of telomere by nucleotide addition; 4. completion of synthesis of telomere repeat and dissociation of telomerase. The dotted arrow represents the possible path of telomerase dissociation from primer. TERC, telomerase RNA component; TERT, telomerase reverse transcriptase; Big dotted cycle, nucleus; Small rectangle, template site; Small ellipse, catalytic subunit.

The mechanism of telomerase activity in synthesizing and lengthening telomeres is as follows: (1) Telomerase is recruited to the telomeric DNA by interacting with a protein called TPP1, which is part of a complex of telomere-binding proteins called shelterin [53]. Other proteins in the complex that are involved in the recruitment of telomerase include TRF2, POT1, and TIN2. TRF2 is a telomere-binding protein that protects the telomere from degradation and helps to recruit telomerase. POT1 is another telomere-binding protein that helps to position telomerase at the end of the telomere. TIN2 is a protein that interacts with both TRF2 and POT1 and helps to stabilize the telomerase complex at the telomere [54]; (2) Telomerase recognizes and binds to the TERC sequence, a repetitive DNA sequence at the 3′ end of the telomere. The TERC sequence in humans is TTAGGG [55]; (3) Telomerase uses the TERC RNA as a template to add more telomeric repeats (TTAGGG) to the 3′ end of the telomere, extending it by 6 nucleotides at a time; (4) Telomerase translocates along the newly extended DNA strand and repeats the synthesis process until the telomere reaches a certain length. The length of the telomere is regulated by various factors, such as the expression and activity of telomerase, the availability of TERC, and the feedback mechanisms of the shelterin complex [54]; (5) The newly synthesized strand of the telomere is complementary to the TERC RNA, and has a 3′ overhang that can fold into a loop structure called the T-loop; (6) Telomerase dissociates from the telomere and the single-stranded overhang is processed by other enzymes, such as DNA polymerase and ligase, to form a double-stranded structure [56]; (7) The last step is the synthesis of the lagging strand of the DNA replication machinery, using the extended strand as a template [56]. The mechanism of the telomere elongation pathway by telomerase is illustrated in Figure 2.

## 3. Telomere length

TL is the measure of the number of DNA base pairs that make up the telomere region at the end of a chromosome. Factors contributing to TL are not fully identified; however, genetics and different environmental factors such as lifestyle and diet may have an effect [56]. TL has been intensively researched in a variety of domains, including genetics, biology, epidemiology, and clinical medicine [16, 57, 58]. It has been proposed as a biomarker of biological aging and age-related disorders [59, 60]. According to numerous studies, TL shortening raises the risk of age-related illnesses such as cancer, cardiovascular diseases, and neurological disorders [61, 62]. For instance, studies indicated that shorter TL was linked to a higher risk of cardiovascular disease and mortality in a sizable sample of middle-aged and older persons [63, 64]. The relationship between TL and lifestyle factors such as smoking and obesity has been investigated [65, 66]. However, a meta-analysis of 18 longitudinal cohorts found that smoking does not accelerate leukocyte telomere attrition in adults [67]. Obesity was linked to shorter TL in both men and women, with contradicting results about the degree of association in different studies [68]. Some studies have found that obesity has a stronger effect on TL in women than in men [69, 70], while other studies have reported no effect on sex differences [65, 71]. Several studies have examined the impact of stress, exercise, and food on TL [72-74]. Additionally, TL has been investigated in relation to psychological and behavioral elements. Long-term psychological stress was connected to shorter TL in a group of healthy women [75]. In the study, TL and telomerase activity were evaluated in PBMCs from 58 healthy premenopausal women who were either mothers of healthy children or mothers of chronically ill children. Mothers of chronically ill children reported more stress and had shorter TL and lower telomerase activity than mothers of healthy children. After controlling for age, body mass index (BMI), and education, perceived stress and chronic stress were found to be negatively correlated with TL and telomerase activity [75]. An increase in TL was reported and linked to mindful meditation, social support, personality traits, and other psychosocial factors in some studies [63, 76, 77]. TL has been described as a potential marker of aging and aging-related diseases, with applications in clinical practice, public health, and personalized medicine. To effectively stop or reverse TL shortening and its harmful effects on health, it requires a clear understanding of the intricate connections between genetic, environmental, and behavioral factors that affect TL dynamics [78].

### 3.1 Factors that regulate TL

It is hard to keep TL in good shape because it needs a careful balance between DNA replication, oxidative stress, and telomere shortening caused by telomerase or ALT-induced telomere extension [79]. Numerous genetic, environmental, and lifestyle factors have been found to have an impact on TL dynamics.

#### 3.1.1 Genetics.

TL is determined mainly by the level of activity of the enzyme, telomerase. Numerous genetic factors, such as mutations in telomerase-encoding gene components or telomere-binding proteins, affect telomerase activity [80]. The proportion of TL variance that has genetic roots is the heritability of TL. Previous research revealed TL heritability values ranging from 34% to 82%, depending on the methodologies and populations studied [79]. A meta-analysis of six large cohort studies with a total of 19,713 participants discovered that TL has a high and consistent heritability estimate of 0.70 [81]. TL heritability can be attributed to two possible sources: inherited variation in non-telomeric regions (e.g., single nucleotide polymorphisms (SNPs) that effect telomere maintenance) and variability in TL in gametes which implies that TL can be passed down from parents to offspring, but the mechanism of inheritance is unclear [79]. Some studies have revealed that the maternal contribution to offspring TL is greater than the paternal contribution, presumably due to mitochondrial DNA or X chromosome effects [82-84]. Other studies have found a paternal age effect, which means that older fathers have offspring with longer TL, presumably due to higher telomerase activity or recombination events during spermatogenesis [85, 86]. Various genes are involved in the regulation of TL including Multiple Endocrine Neoplasia Type 1 (MEN1), Meiotic Recombination 11 Homolog A (MRE11A), RecQ Like Helicase 5 (RECQL5), and Tankyrase 1 (TNKS) [87]. For instance, the catalytic subunit of the telomerase enzyme, which is in charge of attaching telomeric DNA to the ends of chromosomes, is encoded by the TERT gene [45]. It was described that TL and the risk of age-related disorders are related to variations in the TERT gene [88, 89]. TERC, POT1, and TINF2 are other genes involved in TL control by encoding RNA or proteins that forms part of telomerase or the shelterin protein complex [45, 90].

#### 3.1.2 Aging.

During the process of aging, our bodies undergo a gradual degenerative process that manifests through various symptoms. These symptoms can include tissue inflammation, depletion of tissue stem cells, matrix changes, telomere attrition, cellular senescence, and metabolic failure [91]. These changes in cells and tissues are caused by molecular problems in the mitochondria, proteostasis, intercellular communication, nutrition sensing, epigenetics, and DNA repair that lead to genomic instability and damage, such as telomere dysfunction [12, 92]. Mitochondrial dysfunction can result in the buildup of reactive oxygen species (ROS) and oxidative damage to DNA, proteins, and lipids leading to genetic instability [93]. Defective proteostasis can result in the buildup of misfolded or aggregated proteins, which can cause cellular malfunction and injury [93]. Intercellular communication is critical for tissue homeostasis and any disruption can result in persistent inflammation and tissue damage. Nutrient sensing mechanisms including insulin/IGF-1 signaling and mammalian target of rapamycin (mTOR) signaling are critical in controlling metabolism and aging [94]. The disruption of these pathways can result in metabolic malfunction and aging. Epigenetic alterations, such as changes in DNA methylation, can affect gene expression patterns and contribute to aging [95]. Defects in the DNA repair pathway can cause genomic instability and damage [95]. The "end replication problem" and DNA damage from oxidative stress make TL shorter with age and break down the telomeric DNA with each cell division [75, 96]. One of the characteristics of the biological process of aging is age-related TL shortening, which has been seen in a variety of tissues and cell types such as human fibroblasts, leukocytes, and adipocytes [97]. Telomere DNA can be damaged and TL shortening is accelerated by ROS produced by regular cellular metabolism or external stressors [98]. Nutritional supplements containing antioxidants, like vitamins C and E, have been demonstrated in numerous *in vivo* and *in vitro* trials to be protective against TL shortening [99, 100]. Increased oxidative stress, immune cell activation, and TL shortening have all been linked to chronic inflammation [98], which also increases the risk of age-related illnesses and shortens telomeres [31, 98]. Interleukin-6 (IL-6) and tumor necrosis factor-alpha (TNF-α) are examples of inflammatory cytokines that have been demonstrated to decrease telomerase activity and speed up TL shortening by promoting oxidative stress and inflammation in fibroblasts and epithelial cells [101].

#### 3.1.3 Lifestyle and nutrition.

Several lifestyle factors, such as stress, food, physical exercise, and smoking, have been connected to TL dynamics. Some studies found that TL shortening is accelerated by exposure to cigarette smoke, air pollution, or ionizing radiation [65, 102], whereas regular exercise, a healthy diet, and stress management techniques were linked to increased telomerase activity and longer TL [76, 77, 103]. Dietary patterns and specific nutrients have been suggested to influence TL. For instance, a diet rich in fruits, vegetables, whole grains, and some protein has been associated with longer telomeres, while a diet high in processed foods, sugary beverages, and unhealthy fats may be associated with short telomeres [94]. A systematic review was conducted using foods, food intake, dietary patterns, and TL in childhood and adolescence, between the ages of 2 and 18 years [94]. According to the major findings of the review, it was suggested that a high intake of fish, nuts, seeds, fruits, vegetables, green leafy vegetables, cruciferous vegetables, olives, legumes, polyunsaturated fatty acids, and an antioxidant-rich diet may have a good impact on TL [94]. However, TL shortening was linked to a larger intake of dairy products, simple sugars, sugar-sweetened beverages, cereals, mainly white bread, and a diet high in glycemic load [94]. Diet can affect TL and is a modifiable risk factor for chronic disease. In a randomized study of overweight Australian adults, TL was measured by qPCR in samples of lymphocytes, neutrophils, and whole blood after a 12-week nutritional intervention with an almond-enriched diet (AED) to determine any connections between diet quality and TL [58]. 62 overweight or obese people between the ages of 50 and 80 were randomly assigned to either a 12-week isoenergetic nut-free diet (NFD) or an AED for the study. The greater decrease of TL in the NFD group compared to the AED group could be as a result of age, the obese state of the participants, or the short duration of the study. TL was unchanged in lymphocytes, but increased in neutrophils possibly as a consequence of telomere lengthening and slightly decreased in whole blood. The overweight and obese groups experienced similar changes. A significant correlation between changes in food quality scores and changes in lymphocyte, neutrophil, or whole blood TL was not seen [58]. There was no change in the TL of mid- to older Australian adults, but the addition of almonds to the diet led to higher diet quality scores which were assessed based on the Dietary Guideline Index (GDI). It was recommended that future studies examine the effects of more significant dietary changes made over longer time frames [58].

High vitamin intake has been linked to longer TL, while oxidative stress has been linked to shorter TL. Vitamins such as vitamin C and E are antioxidants that protect against oxidative stress and free radicals [104]. The potential to prevent telomere shortening caused by oxidative stress using a multivitamin supplement that included both vitamins and a variety of polyphenolic substances was investigated, using a primary fibroblast cell culture model to test this hypothesis [104]. Together, the results show that a multivitamin supplement combination guards against telomere shortening brought on by oxidative stress in cell culture [104]. A cross-sectional study revealed that there was a positive correlation between vitamin C and TL. The research was conducted using the National Health and Nutrition Examination Surveys (NHANES) database from 1999 to 2002 with 7,094 participants from all races in the United States (51.8% female participants and 48.2% male participants). After correcting for confounding variables such as age, sex, race, BMI, and poverty income ratio (PIR), a multiple linear regression model was used to analyze the link between vitamin C and TL. The 24-hour dietary recall method was used to collect dietary vitamin C intake data. The study found a favorable correlation between vitamin C intake and human TL, which is significant for therapeutic advice on people's healthcare but needs to be confirmed by more thorough and extensive data from other studies [105]. Research was conducted on the role of intake of riboflavin (RF), a water-soluble antioxidant vitamin, on the TL of middle-aged and older American females, especially those with low RF intake. The study revealed that increased dietary intake of riboflavin was significantly associated with longer TL [106]. Zinc is an essential dietary micronutrient that functions as a powerful antioxidant [107]. 24 hours of dietary zinc intake on TL was evaluated on 3,793 US participants aged 45 years and older from 1999 to 2002 using the National Health and Nutrition Examination Survey (NHANES). The research investigated the effect of dietary zinc consumption on TL by assessing the LTL using real-time qPCR. The study revealed that elevated dietary zinc intake was significantly related to longer TL among adults aged 45 years and older in the US. The association was more pronounced in females, obese individuals, and low-energy-intake individuals [107].

Polyunsaturated fatty acids are essential for body functions and growth. Docosahexaenoic acid (DHA) is a polyunsaturated fatty acid that is essential for body functions and growth and is known for its powerful antioxidant and anti-inflammatory properties [108]. A study was conducted to investigate the important properties of DHA in telomere preservation and health promotion, late-generation (G4) telomerase (TERC)-deficient mice were used as a model for telomerase deficiency [109]. The study used a DHA-enriched diet containing 53.0% DHA by weight for 24 months. The result of the study unfortunately showed neither intrinsic nor accelerated aging was prevented in mice by DHA probably as a result of the age of the mice used in the study [109]. However, lifelong dietary DHA supplementation significantly reduced aging phenotypes, senescence markers and telomere attrition in blood leukocytes and numerous tissues, corresponding with decreased galactosidase activity and other senescence-related characteristics. The study concluded that DHA treatment possibly controlled mitochondrial malfunction, which is crucial to the DNA damage response and reduced telomere attrition induced by gamma-H2AX accumulation. The result of the gene and protein expression measurements showed that reduced nuclear factor-B (NF-B), nucleotide-binding domain-like receptor protein 3 (NLRP3), caspase-1, and inhibited ROS accumulation were some of the signs of the impact of DHA on oxidative stress and inflammation, in addition to improved mitochondrial function [109].

## 4. Telomere dysfunction, cellular senescence, and age-related disease

**Figure 3.**
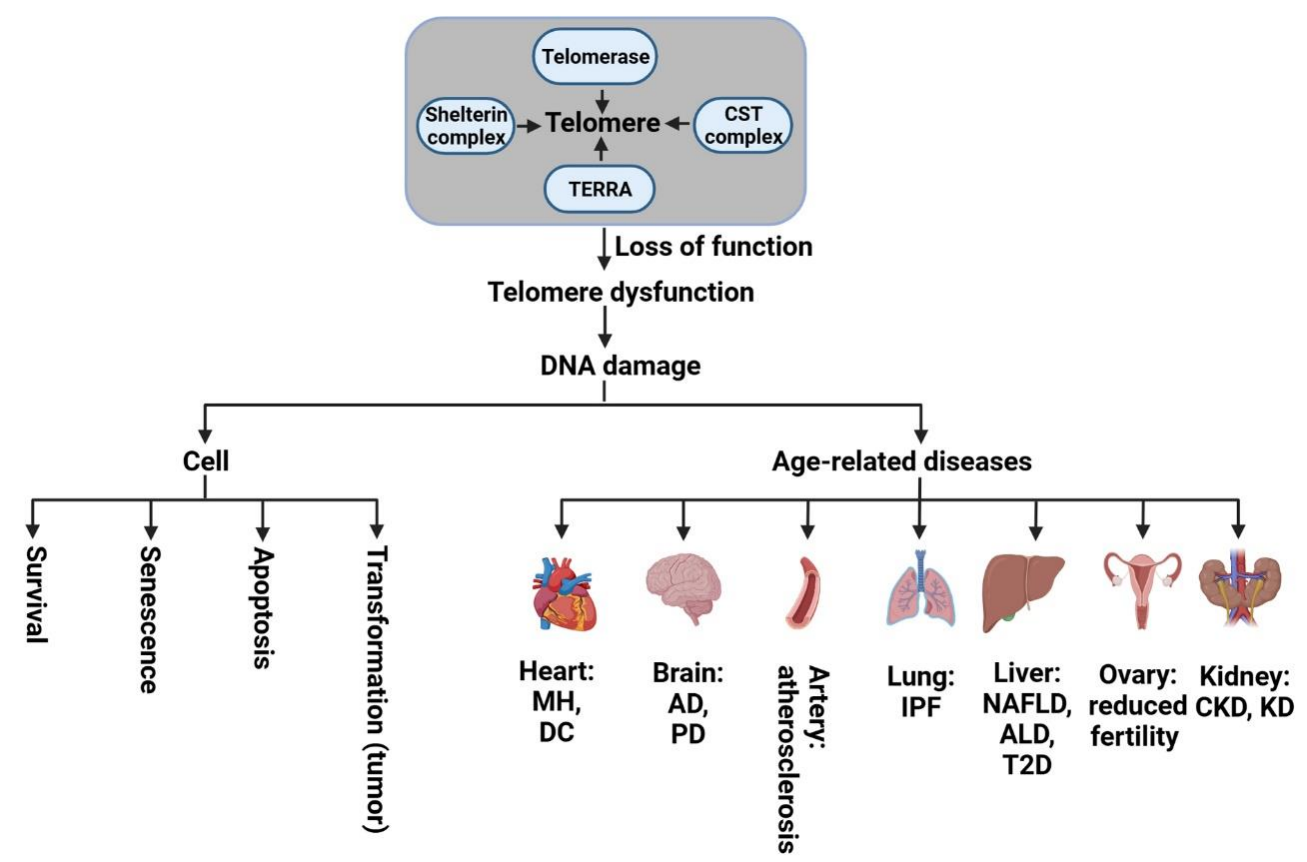
**Telomere dysfunction affects cell fate and age-related diseases**. Telomere regulation involves four factors: telomerase, shelterin complex, CST proteins complex, and TERRA. Dysfunction in any of these factors can cause DNA damage to cells and organs, potentially leading to cell senescence, apoptosis, transformation and age-related diseases. MH, myocardial hypertrophy; DC, dilated cardiomyopathy; AD, Alzheimer’s disease; PD, Parkinson disease; IPF, idiopathic pulmonary fibrosis; NAFLD, non-alcoholic fatty liver disease; ALD, alcoholic liver disease; T2D, type-2 diabetes; CKD, chronic kidney disease; KD, kidney disease. TERRA, telomeric repeat containing RNA; CST complex is composed of three subunits: CTC1, STN1, and TEN1 proteins.

Telomere dysfunction is a state of loss of function of telomere due to factors such as oxidative stress and exposure to ionizing radiation [31], defect in telomere-associated proteins [2, 51, 54], changes in the expression levels of TERRA [49], or mutations in genes encoding proteins required for telomere structure, replication, repair, and length maintenance [28]. When cells in culture approach the Hayflick limit, they stop replicating and enter a non-replicative condition known as senescence/mortality stage 1 (for normal cells) or crisis/mortality stage 2, for oncogenic cells. Cellular senescence is a process where cells stop dividing which can be triggered by various factors, and can lead to age-related disease [37]. One way cellular senescence occurs is through the loss of telomere sequences and damage to the T-loop, which trigger the DDR and result in cell cycle arrest [32]. Studies have revealed that telomere dysfunction and activation of DDR signaling pathways can accelerate cellular senescence and damage various organs in the body such as the heart, brain, artery, lung, liver, ovary, and kidney. These in turn contribute to the development of diseases such as myocardial hypertrophy (MH), dilated cardiomyopathy (DC), Alzheimer’s disease (AD), Parkinson disease (PD), idiopathic pulmonary fibrosis (IPF), non-alcoholic fatty liver disease (NAFLD), alcoholic liver disease (ALD), type-2 diabetes (T2D), chronic kidney disease (CKD), kidney disease (KD), fibrosis, cardiovascular diseases, osteoporosis, and cognitive decline [2, 98, 102, 110-112]. Furthermore, telomere attrition and cellular senescence induced by oxidative stress, DNA damage, and inflammation are related to metabolic imbalances, including insulin resistance, obesity, and dyslipidemia. The implications of telomere dysfunction in DDR, cell, cancer, and age-related disease is illustrated in Figure 3.

### 4.1 Insulin resistance

Insulin resistance, a condition in which the body does not respond normally to insulin, is associated with several conditions, including obesity, cardiovascular disease, NAFLD, and metabolic syndrome [113]. Several studies have linked telomere attrition to insulin resistance, which is a characteristic of T2D and metabolic syndrome. One such study explored the relationship between LTL, expressed by terminal restriction fragment (TRF) length, with insulin resistance, oxidative stress and hypertension in men [114]. The study measured leukocyte TRF length in 327 Caucasian men with a mean age of 62.2 years from the offspring cohort of the Framingham Heart Study. The study found that TRF length was inversely correlated with age and age-adjusted TRF length was inversely correlated with the Homeostatic Model Assessment of Insulin Resistance (HOMA-IR). Compared with their normotensive peers, hypertensive subjects exhibited shorter age-adjusted TRF length, suggesting that hypertension, increased insulin resistance and oxidative stress are associated with shorter LTL and that shorter LTL in hypertensives is largely due to insulin resistance [114]. Another study investigated the relationship between relative LTL and the risk of glycemic progression in patients with T2D [115]. Using two-sample Mendelian randomization analysis, the study analyzed data from 5,349 Chinese patients with T2D from the Hong Kong Diabetes Register (mean age = 57.0 ± 13.3 years; mean follow-up = 8.8 ± 5.4 years) and found that shorter LTL was significantly associated with an increased risk of glycemic progression in individuals with T2D independent of established risk factors [115].

### 4.2 Obesity

Telomere attrition has consistently been linked to obesity, especially abdominal obesity [116, 117]. A study analyzed data from 647 women ages 35 to 74 years in the United States and Puerto Rico (2003-2004) to examine the association between current and past anthropometric characteristics and TL in blood. The study found that higher current BMI and hip circumference were inversely associated with TL. Higher BMI in the 30s was associated with shorter TL among women with ages > 40 years. Weight gain since the age of 30 and weight cycling were also inversely associated with TL. When current BMI and BMI at ages 30 to 39 years were considered together, the most marked decrease in TL was found for women who had overweight or obese BMI at both time points [118]. After controlling for age, sex, and other metabolic characteristics, a systematic review and meta-analysis of studies on the relationship between BMI and LTL identified 29 studies, of which 16 were eligible for meta-analysis, including two longitudinal studies. The majority of studies reported an inverse relationship between BMI and TL. For cross-sectional studies, the pooled estimates for correlation and regression coefficients were - 0.057 and - 0.008, respectively, suggesting a biologically plausible inverse association between BMI and LTL in adults [119]. Additionally, weight loss interventions increased TL in obese people [103].

### 4.3 Dyslipidemia

Dyslipidemia, a condition characterized by high levels of LDL-C and triglycerides and low levels of HDL-C is associated with telomere attrition [120]. A study found that higher low-density lipoprotein cholesterol (LDL-C) and lower high-density lipoprotein cholesterol (HDL-C) levels were associated with shorter TL. The study analyzed data from 77 patients with Cushing’s syndrome (CS) and 77 age-, gender-, smoking-matched controls. The study found that dyslipidemic CS patients had shorter TL than non-dyslipidemic subjects. After adjustment for age and body mass index, cured and active CS dyslipidemic patients had shorter TL than non-dyslipidemic CS. Total cholesterol and triglycerides negatively correlated with TL. No difference in TL according to the presence of other individual cardiovascular risk factors (hypertension, diabetes mellitus, obesity) were observed in CS or in the control group [120].

### 4.4 Metabolic syndrome

Telomere attrition is linked to metabolic syndrome, a collection of metabolic disorders that includes obesity, dyslipidemia, insulin resistance, and hypertension. A longitudinal study using data from 1,808 participants aged 18-65 years from The Netherlands Study of Depression and Anxiety examined whether baseline metabolic syndrome (MetS) components predict TL over time and whether deteriorations in MetS parallel telomere attrition [121]. The study found that higher baseline waist circumference and glucose, and lower high-density lipoprotein (HDL) cholesterol were consistently associated with shorter TL over follow-up. Greater 6-year increase in waist circumference was associated with larger telomere attrition, and similar but nonsignificant associations were observed for a larger increase in triglycerides and glucose levels. The study suggests that metabolic dysregulation is associated with shorter telomeres over two time points [121].

### 4.5 Cancer

Cancer is one of the conditions that has been examined most in relation to telomere attrition. Telomere shortening is regarded as an early event in carcinogenesis. TL has been observed to be shorter in cancer cells than in normal cells, and this shortening is thought to contribute to the onset and spread of cancer [122]. In some other cancers, telomeres are longer than normal as a result of the increased telomerase activity [97]. To retain their replication capacity and avoid cell death, some cancer cells can activate the enzyme telomerase, which lengthens telomeres. This process can cause abnormally long telomeres and support the development and proliferation of tumors [97]. Additionally, some telomere-related genetic mutations such as in the human telomerase gene TERT promoter and in genes involved in the alternative lengthening of telomeres pathway, such as ATRX chromatin remodeler (ATRX) and death domain associated protein (DAXX), have been linked to a high risk of developing cancer [123, 124].

### 4.6 Neurodegenerative diseases

TL is associated with the risk of age-related and neurodegenerative diseases such as Alzheimer’s disease and Parkinson's disease [125]. According to a study by Anitha et al., telomere shortening in neurons can impair their function and promote cell death, contributing to disease progression. The study suggests that TL of chromosomes in white blood cells was associated with fewer brain markers of neurodegeneration and a lower risk of dementia [61]. Also, a recent study suggests that reduced TL in peripheral blood was associated with illnesses of aging phenotypes, such as dementia, and DNA repair deficit disorders, such as ataxia telangiectasia (AT) [126]. The study found that relative TL in dementia was measured as relative telomere copy/single copy gene ratios. Individuals with Huntington's disease had the lowest relative telomere copy/single copy gene ratio (0.21), followed by AT (0.31) and dementia (0.48) [126].

### 4.7 Cardiovascular disease (CVD)

CVD is associated with telomere attrition. Shortening of TL is related to an increased rate of CVD, such as coronary artery disease, heart failure, and stroke [111]. Although the precise mechanisms underlying this association are unknown, endothelial dysfunction, inflammation, and oxidative stress may all play a role [57]. Other diseases such as chronic obstructive pulmonary disease (COPD) [127], and HIV/AIDS [128], have been linked to telomere shortening. According to Savage et al., patients with COPD exhibit shorter telomeres in circulating leukocytes compared to healthy individuals as a result of the induction of systemic inflammation, whereas in the study by Effros et al., the suggested mechanism by which HIV disease was associated with shortened telomeres in the expanded CD28-CD8^+^ cell subset was replicative senescence [127, 128]. Other studies show that subclinical infection with specific pathogens, including cytomegalovirus (CMV), Epstein-Barr virus, human immunodeficiency virus (HIV), hepatitis C virus, malaria and Helicobacter pylori, may accelerate telomere attrition [129, 130].

In contrast, longer TL is associated with various disorders. For instance, compared to controls of the same age, people with Down syndrome were found to have longer telomeres [131]. A study by Armanios et al., on 17 people with POT1 mutations (which result in excessively long telomeres) found that 15 of the 17 participants with the mutations had neoplasms, that ranged from benign to cancerous [132]. Another recent study shows that 8 participants had different types of skin cancer: melanoma, 7 had thyroid neoplasms, and 2 had malignant glioma. In addition, five of the participants with the POT1 mutations had different types of blood-related cancers, and 8 of 12 (67%) people analyzed also had an age-related blood condition called clonal hematopoiesis of indeterminate potential (CHIP), which has been linked to an increased risk for blood and other cancers [133].

## 5. Telomere length and inheritance

TL is an inherited trait that is subject to both genetic and environmental influences [134]. Due to their ability to fend against age-related illnesses and lengthen lifespan expectancy, long telomeres are favorable [135]. Recent research indicates that TL is a hereditary property and that the father's age at conception may be a significant factor in determining TL in offspring [86, 136, 137]. On the other hand, some studies imply that TL is correlated with maternal age at conception [33, 138, 139].

### 5.1 Paternal inheritance of telomere length

While TL has been traditionally believed to be primarily maternally inherited [138], recent studies have shed light on the potential role of paternal inheritance in TL regulation. A positive correlation between a father’s age at conception and offspring TL has been shown in several studies [86, 136]. As sperm TL increases with age, this finding offers indirect evidence in favor of the paternal effect. Theoretically, older fathers at conception will transmit to the zygote their longer sperm telomeres. As a result, the offspring will inherit longer telomeres, which may manifest in blood cells in later life.

Contrary to the work indicating an X chromosome-linked inheritance, wherein the authors found that TL was significantly associated with X chromosome inactivation status in females, but not in males when they measured terminal TRF length in white-blood-cell DNA taken from individuals from the family-based cohort of the Flemish Study on Environment, Genes, and Health Outcomes [140]. Larefalk et al. found no obvious correlation between mothers and offspring regarding mononuclear cells (MNC) telomeres; instead, a statistically significant association between TL comparing father-son and father-daughter pairs was found. However, no correlation was observed between mother-daughter or mother-son. The father-offspring correlation was highly significant (P < 0.0001), in contrast to mother-offspring (P < 0.361). This finding suggests that paternal inheritance is a contributing factor to TL [38]. A study by Njajou et al. on an Amish population in Pennsylvania, USA, revealed that offspring TL is significantly correlated with paternal (r = 0.46), but not maternal TL (r = 0.18) [141]. Such a pattern suggests a paternal effect on TL variation and inheritance. Also, the observation of a link between the daughters' TL and paternal lifespan but not maternal lifespan by the study further clarifies the paternal inheritance of TL [141]. These findings suggest an imprinting mechanism in TL control rather than X-linked trait behavior or a maternal effect. Another study investigating blood cell TL in 962 individuals with an age range between 0 and 102 years and analyzing TL correlations between parent-child pairs in different age groups and between grandparent-grandchild pairs found a highly significant correlation between the father's and the child's TL was observed independent of the sex of the offspring, whereas they observed weaker correlations for mothers and the child’s TL [142]. The study also observed a positive TL correlation for grandparent-grandchild pairs. These findings indicate that fathers contribute significantly stronger to the TL of the offspring compared with mothers. In the study by Xie et al. [60], TL was measured in both parents and their offspring, revealing a significant correlation between paternal TL and that of their offspring (r = 0.26, p < 0.05). Furthermore, a study by Leibel et al. [143] examined the association between paternal age and TL in offspring, demonstrating a negative relationship (p = 0.006). In summary, these several findings suggest that TL is significantly paternally inherited.

### 5.2 Mechanisms of paternal telomere length inheritance

The exact mechanisms underlying the transmission of TL from fathers to offspring remain unclear. Several hypotheses have been proposed, including genetic and epigenetic factors [142, 144]. Genetic factors refer to the presence of specific telomere-related genes on the father’s chromosome or autosomes that influence TL regulation. One suggested mechanism for this is that the sperm cells with longer telomeres are preferentially selected for fertilization, resulting in offspring with longer telomeres, while a second mechanism is that the sperm cells with shorter telomeres undergo more rapid telomere shortening during embryonic development, resulting in offspring with longer telomeres [145]. Epigenetic factors encompass modifications to DNA and histones that regulate gene expression without altering the underlying genetic code. Epigenetic modifications, such as DNA methylation and histone acetylation, have been implicated in TL regulation and may mediate paternal inheritance effects. For example, DNA methylation of telomeres has been closely linked to telomere shortening. Therefore, fathers with higher levels of DNA methylation at telomeres are more likely to pass on this epigenetic mark to their offspring, which could lead to shorter telomeres in the offspring [146, 147]. Findings from another study demonstrate a positive relationship between a father's age at the time of the birth of his child and the length of telomeres in the child’s leukocytes [148]. It was hypothesized that longer LTL in children may be related to older paternal age. This suggests that the father's age at conception may have an impact on TL in leukocytes, a marker of cellular aging processes in the developing immune system [148].

### 5.3 Maternal inheritance of telomere length

Maternal inheritance of TL has been observed in a variety of species, including birds, zebrafish, and humans, with studies also suggesting that TL is maternally inherited [81, 138, 139]. One study found a negative correlation between maternal age and TL in newborns [140], while another study reported a positive correlation between maternal age and TL in adult offspring [86]. The study by Staessen et al. investigated whether TL was influenced by the sex of the parent who transmits the sex chromosome [140]. The authors examined TL in lymphocytes from a random sample of families living in a geographically defined area of northern Belgium. The results of the study suggest that TL may be inherited maternally and linked to the X chromosome. Broer et al. conducted a meta-analysis of TL in 19,713 subjects from 18 studies to investigate the heritability and inheritance patterns of TL [81]. The study found that TL is highly heritable with a heritability estimate of 0.68. The inheritance pattern was found to be stronger in mothers than in fathers, with a maternal heritability estimate of 0.71 compared to a paternal heritability estimate of 0.46. Additionally, the study found a negative correlation between TL and paternal age [81]. Another study analyzed whole genome sequencing data from the Genome of the Netherlands (GONL) project to estimate TL and investigate inheritance patterns of telomeres and genetic variations affecting their length [139]. The study found that parental TL, particularly those of the mother, were stronger predictors of offspring TL than age, which has a weaker effect. Maternal age at conception was positively associated with offspring TL, while the association between paternal age at conception and TL was not observed in this study. The study suggests that the observed correlations between TL and parental TLs and maternal age at conception may reflect Mendelian inheritance of telomeres or inheritance of genetic factors regulating TL. Recently, we explored the associations of TL with age, gender, and clinical variables, and tested the parental effects on TL variation in 1031 Chinese individuals aged between 12 and 111 years, including 108 families with parents and their offspring. We found that TL was shortened with age, however, TL was better maintained in females than males [149]. The study found a robust association of TL between mother and offspring, but not between father and their offspring, indicating that TL is maternally inherited [149]. Other studies have investigated the relationship between maternal TL and offspring health. One study found that a shorter maternal TL was associated with an increased risk of preterm birth [78]. Another study reported an association between shorter maternal TL and increased autism severity in children [150]. These findings suggest that maternal TL may have implications for offspring health and contribute to certain conditions such as preterm birth and autism spectrum disorder. In a previous study by Pascoe et al., the researchers examined TL in children and investigated the potential association with maternal age [151]. The findings indicated that TL was significantly shorter in children born to older mothers suggesting that maternal age may indeed play a role in TL inheritance.

### 5.4 Mechanisms of maternal inheritance of telomere length

The underlying mechanisms for maternal inheritance of TL are not yet fully understood, but several hypotheses have been proposed. One possibility is that mitochondrial DNA (mtDNA), which is inherited exclusively from the mother, plays a role in telomere inheritance. A study found that mtDNA haplogroups were associated with TL in humans, supporting the involvement of mtDNA in TL regulation [152]. Tan et al., did a study on how genetics, socioeconomic status, and exposures while the mother was pregnant affected her nutrition, cardiometabolic health, and mental health, as well as the health of her baby [106]. The association between maternal TL and antenatal maternal health was also studied. They showed that longer maternal TL was positively associated with newborn TL. Mothers with higher anxiety scores, elevated fasting blood glucose, lower plasma insulin-like growth factor-binding protein 3 and vitamin B12 levels, and active smoking status during pregnancy showed a higher risk of giving birth to offspring with shorter TL [106]. The adrenal gland produces glucocorticoids, which are steroid hormones, in response to stress. Synthetic glucocorticoid dexamethasone may stimulate long-term stress in primary amnion epithelial cells leading to senescence or cellular aging [153]. According to a study, treatment with dexamethasone causes these cells to enter senescence through a process that involves both telomeres and the p21 protein. This suggests a possible method by which dexamethasone influences the aging and operation of these particular cells in the amniotic membrane [153]. Several non-human studies such as in birds have highlighted the effect of glucocorticoids on TL inheritance [154, 155]. In a study on nestlings of an altricial passerine bird in natural populations, a positive relationship was found between brood size and baseline corticosterone (CORT) levels and a strong negative correlation between baseline CORT and TL [155]. In another study with wild great tit nestlings during the energy-demanding early growth period, Casagrande et al. found that nestlings with higher baseline corticosterone had shorter telomeres and a higher mitochondrial metabolic rate, and that these nestlings showed increased mitochondrial proton leak, leading to a decreased ATP production efficiency [154].

Also, the hormone cortisol produced by the mother during pregnancy may affect the telomere of the newborn. Studies by Enlow et al., and Ensminger et al., indicated that the length of babies' telomeres may be influenced by the mother's cortisol levels during pregnancy [82]. This impact differs depending on the newborn's sex, according to evidence from the study. This shows a relationship between maternal stress, cortisol, and the molecular mechanisms affecting TL in infants that is particular to sex [82, 156]. Another study showed that TL was shorter in girls between the ages of 9 and 16 who were exposed to gestational diabetes during their mothers' pregnancies [157]. According to the study, gestational diabetes, a disorder that causes high blood sugar levels during pregnancy, may cause telomere shortening in the progeny, especially in females. This shows that gestational diabetes may have long-term effects, even years after delivery, on the cellular aging of female children [157].

In one study, the variables influencing infant TL variation differed according to sex. Maternal TL, mental health, and plasma vitamin B12 levels were the strongest positive predictors of variation in infant TL in females, while paternal age, mother education, and metabolic health were the best positive predictors of variation in newborn TL in males [106]. Mother's TL was linked to her nutritional state and metabolic health, which may have an impact on offspring TL over generations. These results offer insights into the heritable and environmental components and their respective contributions to the early programming of longevity and the first setting of TL. Hence, offspring TL vary based on sex, parental age, and metabolic health of their parents. Another study found that metformin, an anti-diabetic drug, and insulin treatment can stop the shortening of telomeres in the placentas of male infants who were exposed to maternal diabetes during pregnancy [158]. According to the study, metformin and insulin treatments may help maintain TL in the placentas of male kids whose pregnant mothers had diabetes. This suggests that these treatments may have a protective effect on the cellular aging of the placenta in response to maternal diabetes, particularly in male newborns, and that high levels of blood glucose are unhealthy [158]. In addition, epigenetic modifications, specifically DNA methylation of CpG islands in gene promoters are another possible mechanism mediating the maternal effect of TL inheritance in offspring. According to the study by Sergio Andreu-Sánchez and colleagues, the association of maternal age with TL is mediated by decreased methylation of CpG islands located in the promoter of SOX11, with up to 28% of the effect mediated through methylation of SOX11 [110].

### 5.5 Summary of paternal and maternal TL inheritance

The heritability of TL has been demonstrated in numerous studies, that genetic factors play a considerable role in the difference in TL between individuals. Studies on identical twins have revealed better TL concordance than those on dizygotic (fraternal) twins, indicating a significant genetic component [81]. Studies conducted on families have also shown familial aggregation, which is the tendency for close relatives to share TL. These results strongly suggest that genetic mechanisms that control TL exist. Inter-individual variability in TL has been linked to variations in genes encoding telomerase components. There are known SNPs in the TERT and TERC genes that can affect telomerase activity and TL maintenance. While others have been connected to shorter telomeres, certain variants have been linked to longer telomeres [159]. Epigenetic modifications such as histone changes and DNA methylation patterns, regulate TL, telomerase activity, and the functionality of genes such as TERT expression [62]. Telomeres may become abnormally long or short as a result of altered epigenetic markers [160, 161]. These epigenetic marks can be altered by environmental circumstances and lifestyle choices, which may have an effect on TL and speed up the aging or advancement of certain diseases. We summarize the studies that indicated that age, health, immunity, and lifestyles of parents correlate with the TL of offspring in Table 1.

**Table 1:** Patterns of TL inheritance.

Research Sample	Population	Relation Type	Heritability	Methods	Result	Ref.
**Maternal Inheritance**						
**Homo-sapiens**	1031 Chinese individual	Clinical related association of TL with age and gender.	-	qPCR	The shortening of TL in the Chinese population may be caused by impaired lipid metabolism, as TL is inherited from the mother.	[149]
**Free-living jackdaws**	-	Cross-fostering of parent-offspring regression	h2	TRF and qPCR-based studies	Mothers and their offspring's TLs are significantly related	[163]
**Homo-sapiens**	61 Attention Deficit Hyperactive Disorder (ADHD)	Relationship between TL and neuro-developmental disorders in childhood (ADHD)	-	qPCR	Children's TL and maternal, but not paternal, characteristics were connected to the hyperactive-impulsive component of ADHD.	[164]
**Homo-sapiens**	19 713 participants	Examining the variations in maternal versus paternal inheritance for TL	-	qPCR	The heritability estimates for TL are very high and stable, and there is evidence that maternal inheritance and paternal age are positively correlated.	[81]
**Paternal Inheritance**						
**Homo sapiens**	Amish families including 365 men and 551 women	Relationship between TL aging and lifespan	h2	qPCR	The relationship between TL in the offspring and paternal TL was more strongly correlated and positively associated.	[141]
**House Sparrow**	-	Relationship between TL of offspring born to an older and younger parent	-	qPCR	Older fathers produced daughters with longer TLs	[165]
**Other**						
**Homo-sapiens**	312 parents-newborn triad with 104/each	The connection between parents-newborn TL and the telomerase gene (TERT) was the focus of the investigation.	-	qPCR, Sanger sequencing, and flow cytometry	The study finds that the repair of infant TL is highly influenced by TERT, parental TL, prenatal maternal health, and immunity.	[166]
**Mice**	3 TERT-mutant families	Relationship between the telomerase-mutated parents and offspring TL	-	Flow-FISH	The research concluded that, in the presence of normal telomerase expression, the parents' TL serves as the offspring's set point for TL.	[167]
**Fish**		Relationship between sperm count, LTL, STL, and the age of the parents at conception.	-	qPCR	Leukocyte and sperm TL have a substantial positive association with maternal age, and there is a significant positive link between paternal age and STL in the offspring.	[168]
**Homo-sapiens**	32 members of an extended family	Relation between TL and inherited bone marrow syndrome caused by mutations in TERC (ADDC)	-	PCR	Regardless of age, peripheral blood cells from members of a family who lack the TER gene have extremely short telomeres.	[169]
**Homo-sapiens**	287 individuals from 41 families	Relationship between TL of 2 generations and newborn	h^2^	qPCR	LTL does not follow a sex-specific inheritance pattern although it is significantly heritable. It may also be altered by mutual environments.	[170]
**Bird**	-	Parental age and color morphs on offspring relative TL	h^2^	qPCR	RTL is inherited and is influenced by the age of the parent, but is not influenced by color morph.	[171]
**House sparrow nestlings**	2746 hours sparrow nestlings	Parent-offspring regressions	h^2^	qPCR	In a free-living bird, TL is a polygenic variable with a low heritability that is significantly influenced by external factors.	[172]
**Homo-sapiens, Mice, and Monkey**	-	Relationship and comparison between TL of blood leukocytes from outbred and inbred mammalian species to humans	-	Florescent staining	Telomeres elongate as a result of inbreeding, and several segregating loci define the TL for a particular species and/or sub-strain genetically.	[173]

**Figure 4.**
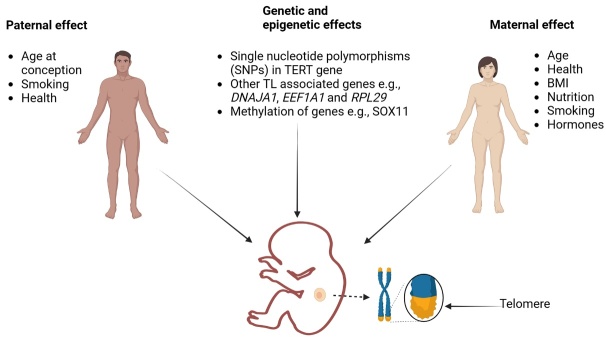
Inheritance factors affecting offspring telomere length (TL). TL is influenced by paternal factors such as age at conception, and maternal factors such as hormones and BMI. Other telomere-related genes and epigenetic mechanisms such as DNA methylation also influence offspring TL. SNPs, single nucleotide polymorphisms; TERT, telomerase reverse transcriptase; DNAJA1, DnaJ heat shock protein family (Hsp40) member A1; EEF1A1, eukaryotic translation elongation factor 1 alpha 1; RPL29, ribosomal protein L29; SOX11, SRY-box transcription factor 11; BMI, body mass index.

Although current research provides increasing evidence that genetics and environmental factors are determinants of telomere attritions leading to age-related disease, further research, samples, and uniform procedures such as DNA extraction from all samples, TL measurement using a standardized method (e.g., quantitative PCR, flow cytometry), statistical analysis to compare TL between individuals, and to assess the mode of inheritance are required to quantitatively determine to what extent TL depends on paternal or maternal inheritance. Figure 4 illustrates the genetic and epigenetic factors that affect TL in offspring.

## 6. Conclusion and future perspectives

According to heritability studies and genetic association studies, stress, diet, exercise, and exposure to toxins are just a few examples of the environmental and genetic factors that affect TL [162]. TL can be affected and have an impact on cellular aging processes due to variations in telomere maintenance genes, such as telomerase components and other telomere-related genes. Insights into the mechanics of aging and age-related disorders can be gained by comprehending the genetic variables that cause TL variation. To have a better understanding of telomere biology and its consequences for human health, further investigation is required to examine the intricate interplay of genetic, epigenetic, and environmental factors.

A better understanding of cellular aging and its effects on human health can be gained by conducting further research on the several factors that have been shown to influence TL. Some potential research areas include TL as a biomarker of aging and disease risk, focusing on establishing the clinical significance of TL measurements in predicting disease susceptibility, response to therapies, and overall health outcomes. The usefulness of LTL for estimating the human biological age, the development of effective tools for slowing down the aging rate, the connection between telomerase and TL during development, their role in carcinogenesis processes, and the consequences of reduced telomerase activity are also potential research areas.

Overall, it is very likely that future studies on the genetic influences of TL will help in understanding the intricate processes involved in cellular aging and illness. Through progress in genomics, functional characterization, and understanding of gene-environment interactions, we can learn more about how genetic factors affect telomere dynamics and come up with ways to promote healthy aging and avoid age-related disorders.
